# Poretti-Boltshauser Syndrome in a Toddler: Novel Neuroimaging Features and Clinical Presentation

**DOI:** 10.7759/cureus.97335

**Published:** 2025-11-20

**Authors:** Shrinivas Radder, Nivedita Radder

**Affiliations:** 1 Diagnostic Radiology/Pediatric Radiology, University of Arkansas for Medical Sciences, Arkansas Children's Hospital, Little Rock, USA; 2 Diagnostic Radiology, University of Arkansas for Medical Sciences, Little Rock, USA

**Keywords:** cerebellar cysts, cerebellar dysplasia, developmental delay, lama1 gene, pediatric neuroimaging, poretti-boltshauser syndrome

## Abstract

Poretti-Boltshauser syndrome (PBS) is a rare autosomal recessive disorder characterized by cerebellar dysplasia with cysts, developmental delay, and ocular abnormalities. We present a one-year-old male with developmental delay, hypotonia, and abnormal eye movements. Brain magnetic resonance imaging revealed extensive cerebellar dysplasia with multiple cortical and subcortical cysts, an enlarged rhomboid-shaped fourth ventricle, and splayed superior cerebellar peduncles. Genetic testing confirmed compound heterozygous pathogenic variants in the LAMA1 gene, both classified as pathogenic according to American College of Medical Genetics and Genomics (ACMG) guidelines. This case highlights the distinctive neuroimaging pattern of PBS, which can guide early diagnosis and appropriate genetic testing. Recognition of these imaging features is crucial for pediatric radiologists, as early diagnosis enables timely intervention with supportive therapies and genetic counseling for affected families.

## Introduction

Poretti-Boltshauser syndrome (PBS) represents a distinctive cerebellar malformation syndrome first delineated as a clinical entity in 2014. The condition results from biallelic mutations in the LAMA1 gene, which encodes laminin alpha-1, a crucial component of basement membranes involved in cerebellar and retinal development [1]. The syndrome manifests with a characteristic triad of cerebellar dysplasia with cysts, non-progressive cerebellar ataxia, and variable intellectual disability, often accompanied by ocular motor apraxia and myopia [2].

The neuroimaging phenotype of PBS provides critical diagnostic clues that can direct genetic testing and avoid diagnostic odysseys for affected families. While cerebellar malformations encompass a heterogeneous group of disorders, the specific combination of imaging findings in PBS creates a recognizable pattern that distinguishes it from other conditions such as alpha-dystroglycanopathies, GPR56-related polymicrogyria, and Joubert syndrome [3]. For comprehensive reviews of cerebellar malformations and their classification, readers are directed to Poretti et al. (2014) and Boltshauser et al. (2015) [3].

Recognition of the PBS neuroimaging phenotype is essential not only for diagnosis but also for efficient use of genetic resources, as it allows for targeted rather than broad genetic testing approaches. We present a case that illustrates the characteristic neuroimaging features of PBS in a young child, emphasizing the importance of pattern recognition in establishing this diagnosis.

## Case presentation

A one-year-old male infant was referred to our pediatric neurology clinic for evaluation of developmental delay and abnormal eye movements. The child was born at term following an uncomplicated pregnancy to non-consanguineous parents. Birth weight was 3.2 kg, and Apgar scores were 8 and 9 at 1 and 5 minutes, respectively. The parents first noticed concerns at approximately four months of age when the infant demonstrated poor visual tracking and delayed motor milestones.

At presentation, the child exhibited global developmental delay with particular impairment in gross motor skills. He had achieved head control at 6 months but was unable to sit independently at 12 months. Physical examination at 12 months revealed normal growth parameters for age, with weight and length tracking along the 25th-50th percentiles according to parental report. Head circumference was not specifically documented, but appeared clinically normocephalic without signs of microcephaly or macrocephaly. Neurological assessment revealed mild generalized hypotonia with preserved deep tendon reflexes. Horizontal nystagmus on rightward gaze and intermittent esotropia were noted. The child moved all extremities equally but with decreased spontaneous movement compared to age-matched peers. When supported, his gait was notably wide-based and unsteady. No dysmorphic features were observed, and systemic examination was unremarkable.

**Figure 1:**
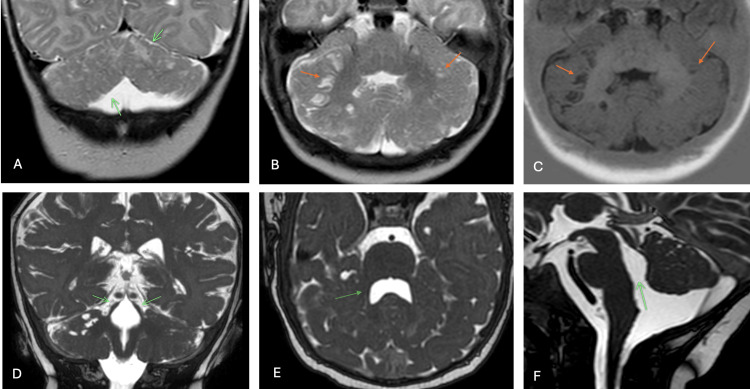
Neuroimaging characteristics of Poretti-Boltshauser syndrome (A) Coronal T2-weighted image demonstrates dysplastic cerebellar architecture with disorganized folial patterns (arrows). (B, C) Axial T2-weighted and T1-weighted sequences reveal multiple small cysts at the cortical-subcortical junction throughout both cerebellar hemispheres (arrows). (D) Coronal T2-weighted image displays an abnormally thin and splayed configuration of the superior cerebellar peduncles (arrows). (E, F) High-resolution T2-weighted sequences in axial (E) and midsagittal (F) planes demonstrate marked enlargement of the fourth ventricle with characteristic rhomboid morphology and blunting of the fastigial recess.

Brain magnetic resonance imaging performed at our institution revealed striking abnormalities of the posterior fossa structures. The cerebellar hemispheres and vermis demonstrated marked dysplasia with extensively disorganized architecture. The normal folial pattern was replaced by anomalous, irregular folia and fissures throughout both cerebellar hemispheres. Multiple small cortical and subcortical cysts were identified bilaterally within the cerebellum, ranging from 2 to 8 millimeters in diameter. The superior cerebellar peduncles appeared thin and splayed, creating a configuration reminiscent of the molar tooth sign, though less pronounced than typically seen in Joubert syndrome. The fourth ventricle was notably enlarged with an unusual rhomboid configuration and loss of the normal pointed appearance of the fastigium. The brainstem appeared mildly elongated but otherwise structurally normal. Supratentorial structures showed no abnormalities, with normal cortical development and myelination appropriate for age. Both globes demonstrated a slightly elongated configuration on sagittal sequences (Figure 1).

Given the distinctive neuroimaging findings suggestive of PBS, targeted genetic testing was performed. Molecular analysis revealed compound heterozygous frameshift variants in the LAMA1 gene, both classified as pathogenic according to American College of Medical Genetics and Genomics (ACMG) criteria. The first variant, a 7-base pair deletion in exon 14 causing a frameshift, met criteria PVS1 (null variant in a gene with an established loss-of-function mechanism), PM2 (absent from population databases, including gnomAD), and PP3 (multiple in silico tools predict deleterious effect with CADD score >30). The second variant, a 10-base pair duplication in exon 24, also resulting in frameshift, similarly fulfilled PVS1 (null variant), PM2 (absent from population databases), and PP3 (computational evidence supports deleterious effect). Both variants are novel and have not been previously reported in the literature or the ClinVar database. The frameshift nature of both variants leads to premature termination codons, consistent with the loss-of-function mechanism established for PBS. Conservation analysis using phyloP and GERP++ showed high conservation scores (>4) at both variant positions across vertebrate species. Parental segregation analysis confirmed biallelic inheritance (PM3), further supporting pathogenicity.

Following diagnosis, ophthalmological evaluation documented mild bilateral myopia (-2.50 diopters). While detailed retinal imaging and formal ERG were not performed at initial assessment, annual ophthalmological surveillance was recommended given the known association of LAMA1 mutations with progressive retinopathy.

The child was enrolled in an early intervention program with physical, occupational, and speech therapy. At the 18-month follow-up, he had achieved supported sitting and was making gradual developmental progress, though he remained below age-appropriate milestones.

## Discussion

This case exemplifies the characteristic neuroimaging phenotype of Poretti-Boltshauser syndrome, demonstrating the value of pattern recognition in guiding genetic diagnosis. The combination of cerebellar dysplasia with cysts, enlarged rhomboid-shaped fourth ventricle, and splayed superior cerebellar peduncles creates a distinctive imaging signature that, when recognized, can expedite diagnosis and avoid extensive metabolic and genetic testing [4]. The key imaging features that distinguish PBS from other cerebellar malformations are summarized in Table 1.

**Table 1:** Neuroimaging differential diagnosis of cerebellar dysplasia with cysts

Feature	PBS	Joubert Syndrome	α-Dystroglycanopathies
Cerebellar cysts	Multiple, cortical-subcortical, bilateral	Absent	Multiple, often subcortical
Cyst distribution	Superior vermis, posterior hemispheres	N/A	Variable, often inferior
Fourth ventricle	Enlarged, rhomboid-shaped	Bat-wing shaped	Variable, often normal
Superior cerebellar peduncles	Thin, mildly splayed	Thick, horizontal (molar tooth sign)	Normal
Vermis	Dysplastic, inferior hypoplasia	Hypoplastic with midline cleft	Dysplastic
Supratentorial findings	Normal	Variable (25-30%)	Cobblestone cortex, ventriculomegaly
Brainstem	Mild elongation	Deep interpeduncular fossa	Pontine hypoplasia common
Associated features	Normal muscle/CK	Possible organ involvement	Elevated CK, muscle weakness

The rhomboid configuration of the fourth ventricle in PBS is distinctive and differs from the bat-wing shape seen in Joubert syndrome or the normal/compressed appearance in α-dystroglycanopathies. This rhomboid shape results from specific developmental disruption of the fastigium and lateral recesses, creating a diamond-like appearance on axial images that is virtually pathognomonic for PBS when combined with cerebellar cysts. The distribution of cerebellar cysts also provides diagnostic specificity. In PBS, cysts preferentially involve the superior vermis and posterior-superior hemispheres, reflecting the role of LAMA1 in basement membrane integrity during late cerebellar development [5]. The superior cerebellar peduncles in PBS show mild splaying without the thickening and horizontal orientation that creates the molar tooth sign in Joubert syndrome. This subtle difference is crucial: PBS peduncles remain thin and maintain some vertical orientation, whereas Joubert syndrome shows thick, horizontally oriented peduncles with a deep interpeduncular fossa [6].

The distinctive neuroimaging pattern of cerebellar dysplasia with cysts, enlarged rhomboid fourth ventricle, and splayed superior cerebellar peduncles establishes a strong presumptive diagnosis of PBS that warrants genetic confirmation. In current clinical practice, these characteristic findings would typically prompt targeted LAMA1 sequencing or inclusion of LAMA1 in next-generation sequencing panels for cerebellar malformations. The genetic testing in our case confirmed what the imaging strongly suggested, reinforcing that pattern recognition can effectively guide genetic testing strategies.

The identification of compound heterozygous frameshift variants in our patient adds to the expanding molecular spectrum of PBS. Both variants result in frameshift mutations leading to premature protein truncation, consistent with the predominance of loss-of-function mutations reported in PBS [7]. The presence of two distinct frameshift mutations, rather than a homozygous mutation, reflects the genetic heterogeneity observed in PBS and is typical of cases from non-consanguineous families. The molecular heterogeneity observed in PBS, with over 30 different pathogenic LAMA1 variants reported to date, contrasts with the relatively consistent clinical and neuroimaging phenotype [8].

Ophthalmological monitoring in PBS

We recommend baseline ophthalmological assessment at diagnosis, including funduscopy and electroretinogram (ERG) when feasible, followed by annual surveillance. Monitoring should include annual dilated fundus examination and refraction, ERG every two to three years or on clinical deterioration, optical coherence tomography (OCT) when age-appropriate to assess photoreceptor integrity, and increased frequency (six-monthly) if retinal changes are detected. Early detection enables timely refractive correction and identification of rare complications such as retinal detachment. While no specific treatment exists for PBS-related retinopathy, optimizing visual function through appropriate optical correction remains important [9].

Clinical take-home message

Recognition of the PBS neuroimaging triad - cerebellar dysplasia with multiple cortical-subcortical cysts, enlarged rhomboid-shaped fourth ventricle, and thin, splayed superior cerebellar peduncles - should prompt immediate consideration of Poretti-Boltshauser syndrome and targeted LAMA1 gene sequencing. The rhomboid configuration of the fourth ventricle combined with bilateral cerebellar cysts is virtually pathognomonic for PBS, distinguishing it from Joubert syndrome (which lacks cysts and shows thick horizontal superior cerebellar peduncles forming the molar tooth sign) and α-dystroglycanopathies (which typically present with supratentorial abnormalities and elevated creatine kinase). Early recognition of this imaging pattern enables prompt genetic diagnosis, avoiding extensive metabolic testing while facilitating timely intervention with appropriate developmental therapies and accurate genetic counseling regarding the 25% recurrence risk.

## Conclusions

Poretti-Boltshauser syndrome presents with a distinctive neuroimaging phenotype that enables early diagnosis when recognized by pediatric radiologists and neurologists. The combination of cerebellar dysplasia with cysts, enlarged rhomboid-shaped fourth ventricle, and splayed superior cerebellar peduncles should prompt genetic testing for LAMA1 variants. Early diagnosis facilitates timely intervention with supportive therapies and provides families with accurate prognostic information and recurrence risk counseling.

## References

[1] Aldinger KA, Mosca SJ, Tétreault M (2014). Mutations in LAMA1 cause cerebellar dysplasia and cysts with and without retinal dystrophy. Am J Hum Genet.

[2] Micalizzi A, Poretti A, Romani M (2016). Clinical, neuroradiological and molecular characterization of cerebellar dysplasia with cysts (Poretti-Boltshauser syndrome). Eur J Hum Genet.

[3] Poretti A, Boltshauser E, Doherty D (2014). Cerebellar hypoplasia: differential diagnosis and diagnostic approach. Am J Med Genet C Semin Med Genet.

[4] Poretti A, Häusler M, von Moers A (2014). Ataxia, intellectual disability, and ocular apraxia with cerebellar cysts: a new disease?. Cerebellum.

[5] Boltshauser E, Scheer I, Huisman TA, Poretti A (2015). Cerebellar cysts in children: a pattern recognition approach. Cerebellum.

[6] Vilboux T, Malicdan MC, Chang YM (2016). Cystic cerebellar dysplasia and biallelic LAMA1 mutations: a lamininopathy associated with tics, obsessive compulsive traits and myopia due to cell adhesion and migration defects. J Med Genet.

[7] Romani M, Micalizzi A, Valente EM (2013). Joubert syndrome: congenital cerebellar ataxia with the molar tooth. Lancet Neurol.

[8] Radmanesh F, Caglayan AO, Silhavy JL (2013). Mutations in LAMB1 cause cobblestone brain malformation without muscular or ocular abnormalities. Am J Hum Genet.

[9] Edwards MM, Mammadova-Bach E, Alpy F (2010). Mutations in Lama1 disrupt retinal vascular development and inner limiting membrane formation. J Biol Chem.

